# Afterdischarges of Spinal Interneurons Following a Brief High-Frequency Stimulation of Ia Afferents in the Cat

**DOI:** 10.3389/fnint.2019.00075

**Published:** 2020-01-24

**Authors:** Abraham Méndez-Fernández, Mayra Moreno-Castillo, Nayeli Huidobro, Amira Flores, Elias Manjarrez

**Affiliations:** Instituto de Fisiología, Benemérita Universidad Autónoma de Puebla, Puebla, México

**Keywords:** interneuros, bistability, afterdischarges, DC shift, spinal cord

## Abstract

Spinal motoneurons exhibit sustained afterdischarges and plateau potentials following a brief high-frequency stimulation of Ia afferents. Also, there is evidence that spinal cord interneurons exhibit plateau potentials. However, to our knowledge, there are no reports about the possible afterdischarge behavior of lumbar spinal interneurons activated by Ia afferents. Given that there are spinal interneurons receiving monosynaptic inputs from Ia afferents, these cells could then be activated in parallel to motoneurons after repetitive muscle stretch. We explored this possibility in cats with a precollicular-postmammillary decerebration. We found that a brief high-frequency stimulation of Ia afferents produces afterdischarges that are highly correlated to a DC slow potential recorded at the cord dorsum. We conclude that in the cat spinal cord, not only the motoneurons but also the interneurons from the superficial and deep dorsal horn produce sustained afterdischarges, thus highlighting the importance of interneurons in the spinal neuronal circuitry. The significance of our finding is that it opens the possibility that the spinal cord interneurons activated by Ia afferents could also exhibit bistability, a relevant phenomenon well-characterized in the motoneurons.

## Introduction

The firing pattern of a spinal motoneuron can alternate between two stable states depending on the immediate previous history of such motoneuron; this is called bistability (Hounsgaard and Kiehn, 1985; Hounsgaard et al., 1988). Functionally, this property allows a motor unit to self-sustain its activity during the performance of long-lasting tonic motor tasks, such as postural tone maintenance (Eken and Kiehn, 1989; Gorassini et al., 1998). Also, under certain pathological circumstances, this ability gets anomalously increased in the motoneurons as it is believed to occur during spasticity after chronic spinal injury (Harvey et al., 2006a,b). A shift into a high-frequency firing state in the motoneurons can be achieved through the high-frequency stimulation of the homonymous muscle stretch receptors and specific brainstem nuclei (Granit et al., 1957; Mori et al., 1982; Crone et al., 1988; Bennett et al., 1998). The integrity of monoaminergic descendent pathways is fundamental for the activation of motoneuron bistable properties (Conway et al., 1988; Kiehn et al., 1996; Perrier and Delgado-Lezama, 2005; Perrier et al., 2013).

The capability to generate afterdischarges is shared by different neurons in the spinal cord. For instance, similarly to motoneurons, a class of spinal interneurons can exhibit sustained discharges after a brief exposure to electrical stimulation to the sensory pudendal nerve of female cats (Cueva-Rolón et al., 2002; Muñoz-Martínez and Delgado-Lezama, 2007). Also, interneuron afterdischarges can be related to the persistence of secondary pain (Price et al., 1978) and the triggering of the scratch pattern (Currie and Stein, 1990). In the same way, the capability to generate plateau potentials is shared by many neurons in the spinal cord (Abbinanti et al., 2012; Husch et al., 2014). For example, motoneurons and interneurons exhibit the property to generate plateau potentials (Russo and Hounsgaard, 1994, 1996; Abbinanti et al., 2012; Husch et al., 2014). Furthermore, the mechanisms that underlie plateau generation in both sets of spinal neurons are substantially similar (Hounsgaard and Kjaerulff, 1992; Svirskis and Hounsgaard, 1997; Abbinanti et al., 2012; Husch et al., 2014).

The present study aimed to examine whether a brief high-frequency stimulation of Ia afferents produces afterdischarges in spinal interneurons as was previously found for the spinal motoneurons (Hounsgaard et al., 1988; Lee and Heckman, 1998). Since there are spinal interneurons receiving monosynaptic and polysynaptic contacts from Ia afferents (Kanda, 1972; Brown, 1981; Harrison and Jankowska, 1985; Bannatyne et al., 2009), it is feasible that these cells are recruited in parallel to motoneurons after repetitive muscle stretch. Our results will highlight the importance of interneurons in the spinal neuronal circuitry.

## Materials and Methods

### Preparation

Experiments were performed in seven adult cats whose weights ranged between 2.3 and 4.5 kg. We performed two types of experiments: (1) recording of the spinal cord dorsum in four cats; and (2) multiunit recording of spinal interneurons in four cats. In one cat, we performed both experimental paradigms. The protocol was approved by the ethics committee (CICUAL-Proyecto-00489) of the Benemérita Universidad Autónoma de Puebla. The guidelines stipulated in the Guide for the Care and Use of Laboratory Animals (Eighth Edition, revised in 2011) and the Mexican regulations (NOM-062-ZOO-1999) were carefully followed. Before surgery, anesthesia was induced by a volatile mix of isoflurane (3%–4%) and oxygen (96%–97%). After tracheostomy, anesthesia was maintained *via* a tracheal cannula until decerebration. Atropine (0.05 mg kg^−1^) and dexamethasone (2 mg kg^−1^) were administered at the beginning of the procedures. The radial vein and the common carotid artery were cannulated for liquid administration and blood pressure monitoring, respectively. We maintained the blood pressure between 80 and 120 mmHg by the administration of Dextran and glucose (5%) isotonic solution. Bicarbonate (100 mM) and glucose (5%) solution were delivered at a constant flux (5 ml h^−1^) throughout the whole experiment *via* the common carotid artery. The temperature was kept inside a physiological range through a heating pad over which the animal was laid and a radiant heat lamp. The decerebration method consisted of a precollicular-postmammillary transection. After the decerebration, anesthesia was gradually suspended. At the end of the experimental procedures, a lethal dose of pentobarbital sodium was administered intravenously.

### Recording of the Spinal Cord Dorsum

**Figure 1:**
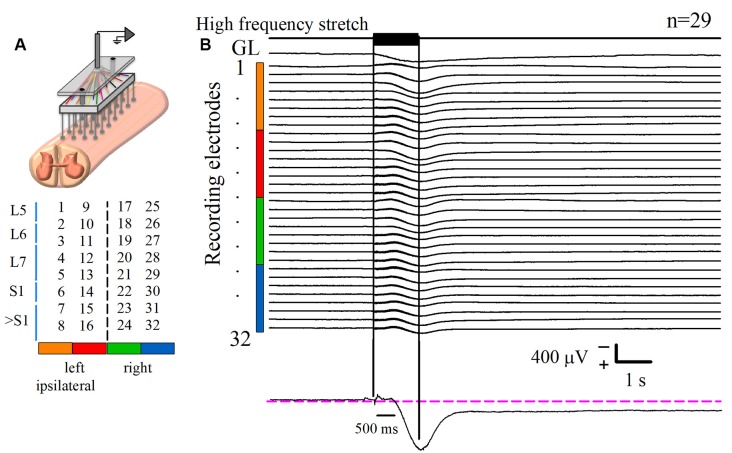
Recording of the DC potential on the spinal cord dorsum in response to a high-frequency stretch stimulus applied to the Achilles tendon. **(A)** Scheme of the recording paradigm, which consisted of an array of 32 (8 × 4) Ag-AgCl spherical electrodes positioned over the surface of the lumbosacral spinal cord. The array was coupled to a Synamps amplifier system in DC mode. The midline is represented by the dashed line that divides the two halves of the array. Spinal segments are specified at the left, according to Marcoux and Rossignol (2000). **(B)** The first upper trace is the high-frequency stretch stimulus applied on the left Achilles tendon. The two vertical black lines indicate the duration of such stimulus. The trace labeled with *gastrocnemius lateralis* (GL) is the rectified and integrated electroneurographic (ENG) signal recorded from the *GL* nerve. See how the GL activity experiences a sustained rise following the stimulus. The next 32 traces are the averaged DC potentials recorded from the spinal cord dorsum after several trials (*n* = 29) of stimulation. Such signals were obtained from the electrode array represented in **(A)**; see color code. Note the marked positive DC shift that outlasts the mechanical stimulus duration for 6.4 ± 0.3 s (mean ± standard error). Observe that the sustained slow DC potential in the cord dorsum occurs concomitantly with the GL ENG activity. The recording in the lower panel is a zoom of a slow DC potential.

In four cats, a laminectomy was performed to expose the lumbosacral spinal enlargement. We recorded the activity of the spinal cord dorsum using an array of 32 Ag-AgCl spherical electrodes (diameter of 200 μm), positioned over the surface of the lumbosacral spinal cord (Figure 1A). Such activity was acquired with the Synamps 2 amplifier (CompuMedics NeuroScan) at a sampling rate of 10 kHz and filters in DC mode. Simultaneously, the electroneurographic (ENG) activity was recorded from the *gastrocnemius lateralis* (GL) nerve, employing a bipolar hook silver wire electrode. GL activity was rectified and integrated. Pools were made at the hind limb exposed areas and filled with mineral oil.

### Multiunit Recording of Spinal Interneurons

We also recorded the multiunit activity (MUA) of the superficial and deep dorsal horn interneurons of the L7 segment in four cats. These neurons did not respond to antidromic stimulation of the ventral roots. For this purpose, we employed quartz/platinum-tungsten fiber microelectrodes (1–2 MΩ) coupled with the Minimatrix system (Thomas Recording). Signals were acquired by a Digidata system (Molecular Devices, San Jose, CA, USA) at a sampling rate of 250 kHz. We used the “WaveClus” spike-sorting algorithm (Quiroga et al., 2004) to obtain unitary spikes from the raw signals.

### Mechanical Stimulation of Stretch Receptors

The Achilles tendon of the left hind limb was dissected and coupled to a mechanical stimulator (Chubbuck, 1966). In this form, the mechanical stimulator administered vibrating stimuli to the *triceps surae* muscle. The stimulus consisted of high-frequency (200 Hz) and low-amplitude (200 μm) brief vibration (0.85–1.2 s). The stimulus characteristics ensured activation of the total amount of *triceps surae* Ia afferents (Lucas and Willis, 1974).

### Statistical Analysis

The instantaneous firing rate of the sorted units was measured and grouped into three intervals: “before,” “during,” and “after” the stimulus presentation. Time zero was defined as the instance of the beginning of stimulus presentation. The rates were compared by applying the nonparametric Friedman test and a *post hoc* Wilcoxon signed-rank test (R version 3.3.0, The R Project for Statistical Computing). The Pearson correlation coefficient between the DC signal and the relative counts of interneuronal spike times was computed. In all cases, the threshold for a type I error was set at 0.05.

## Results

Spinal cord dorsum activity was recorded in response to a brief high-frequency repetitive stretch of the Achilles tendon. After the stimulus presentation, the baseline cord dorsum potential recorded over the bilateral lumbosacral spinal cord exhibited a positive and slow DC shift. This slow DC potential was observed with a peak latency of 0.96 ± 0.004 s after the beginning of the stimulation. Figure 1B shows the averaged traces of 29 trials obtained from one cat. To characterize the slow DC potential, a double exponential model was fitted to these signals. A decay of 0.95 of the maximum amplitude was the criterion to determine the end of the DC potential. We found that the slow DC potential outlasted the duration of the stimulus by 6.4 ± 0.3 s (four cats, 108 trials); that is, we observed an “afterdischarge” in the cord dorsum activity due to the Ia afferent activation. More interestingly, this phenomenon occurred during the ENG sustained activity of the GL nerve (Figure 1B). This co-activation of spinal cord dorsum and motor output (GL) activity was seen in four animals.

**Figure 2:**
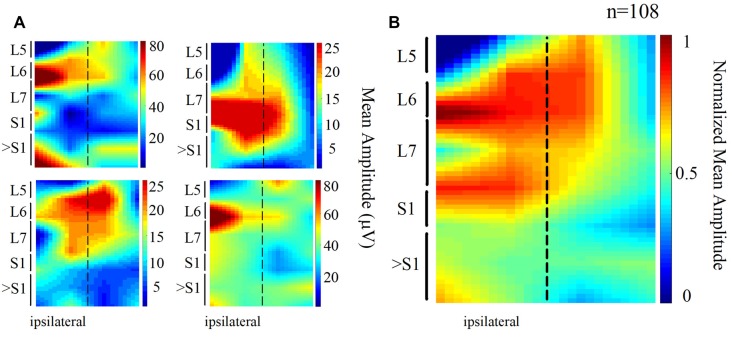
Topographic maps obtained from the spatial distribution of the DC potentials recorded from the cord dorsum. **(A)** Each map was obtained from one animal. Again, the dashed line represents the midline. Larger amplitudes tend to focus at the ipsilateral (left) L6 and L7 segments. **(B)** Grand average of the four normalized maps in **(A)** *n* = 108 trials, four cats.

We also found that the DC potential amplitude depended markedly on the recording site. The spatial distribution of the mean amplitude of the slow DC potential is presented in Figure 2. Each map of Figure 2A was obtained from one animal (four cats), whereas the map of Figure 2B is the grand average of the normalized maps shown in Figure 2A (four cats, 108 trials). In these maps, the dashed line represents the midline. We can observe that the amplitude of the DC potential was higher at the L6–L7 spinal segments ipsilateral to the stimulus presentation. This result was expected in accordance with the reported Ia afferent branching pattern, as discussed later.

**Figure 3:**
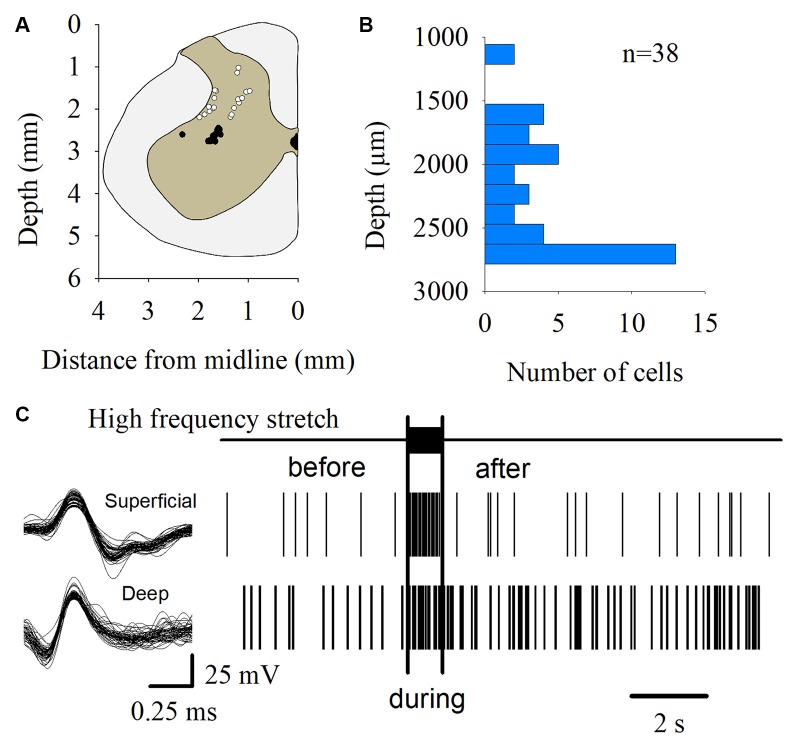
Multiunit recording of lumbar interneurons from the superficial and deep dorsal horn.** (A)** The white and black circles represent the sites from which the multiunit recordings of the spinal neurons were performed. The white circles represent the superficial dorsal horn neurons. The black circles represent the deep dorsal horn neurons. **(B)** Distribution of the depth of the recorded interneurons (*n* = 37) shown in **(A)**. **(C)** Scheme representing the spike-sorting for both the superficial and deep dorsal horn neurons, as indicated. Note that the superficial dorsal horn neurons exhibited a slower firing rate than the deep dorsal horn neurons. The signals from single interneurons were obtained from the raw multiunit activity (MUA). The waveforms illustrated in the left panel are superimposed traces of two interneurons from two different animals. The right panel shows the raster plots of such neurons in response to the high-frequency stretch of the Achilles tendon (uppermost trace and vertical bars).

**Figure 4:**
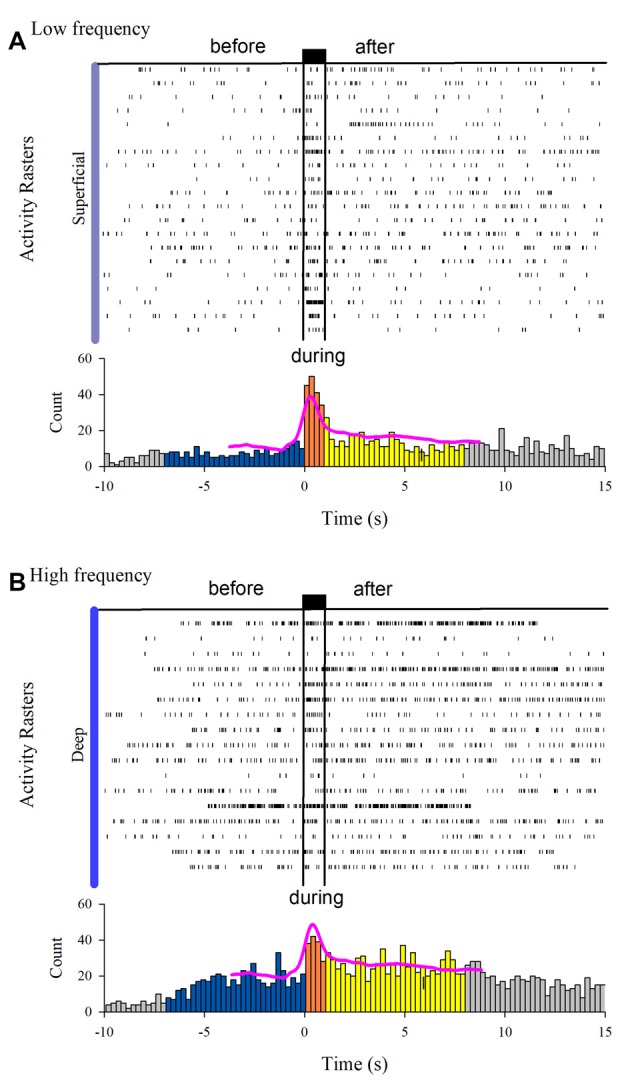
Interneuron afterdischarges of superficial and deep dorsal horn neurons. **(A)** Rasters of spiking activity from 20 superficial dorsal horn interneurons. Note the increase in the activity during and after the stimulation compared to the basal activity before stretch. The uppermost trace depicts the high-frequency stretch stimulus applied to the Achilles tendon. The duration of the stimulus is indicated by the two vertical bars. **(B)** The same as **(A)** but for 17 deep dorsal horn interneurons. The relative count histograms from the activity rasters are illustrated with the superimposed recording of the DC cord dorsum potential.

The MUA was recorded at the L7 spinal segment from the sites drawn as white and black circles in Figure 3A. The distribution of the depth of recording sites is roughly bimodal, with the first mode around 2,000 μm and the second one about 2,800 μm (Figure 3B). In such locations, we found 37 interneurons that responded with unitary firing activity to muscle stretch. We recorded 20 superficial neurons (see white circles in Figure 3A) whose firing activity exhibited a low firing rate (Figure 3C) and 17 deep interneurons (see black with circles in Figure 3A) whose firing activity exhibited a high firing rate (Figure 3C). The pooled activity of both classes of interneurons is displayed in Figures 4A,B, in which each dot represents a single spike and the rows stand for different interneurons. The two black vertical lines indicate the stimulus duration. Sustained afterdischarges were observed after the cessation of the stimulus for up to 7 s; see the rasters of firing activity following the stimulation compared to the basal activity. Also, see the yellow bars in the histograms of Figure 4 in comparison to the blue bars; note that the height of the yellow bars remains well above the basal activity (dashed horizontal line). To quantify these changes, the instantaneous firing rate was measured and compared between the following intervals: “before” (−7 to 0 s), “during” (0–0.93 s), and “after” (0.93–7.93 s). For all of the neurons (37), the mean firing rate was calculated considering the values of all interneurons throughout the corresponding time window. The instantaneous firing rates were time-averaged to obtain mean values of 22.2 ± 0.6 Hz, 27.2 ± 1.2 Hz, and 26.15 ± 0.74 Hz, respectively. We found statistical differences among these intervals (Friedman test, *p* = 0.012). Additionally, a *post hoc* comparison yielded statistical differences when applied to “before” vs. “during” (*p* = 0.002) and to “before” vs. “after” (*p* = 0.014) intervals. Finally, we found a strong correlation between the grand average of the DC potential recorded on the cord dorsum and the relative counts of the interneuronal spikes (Pearson correlation coefficient *r* = 0.78, *p* = 2.9 × 10^−12^).

**Figure 5:**
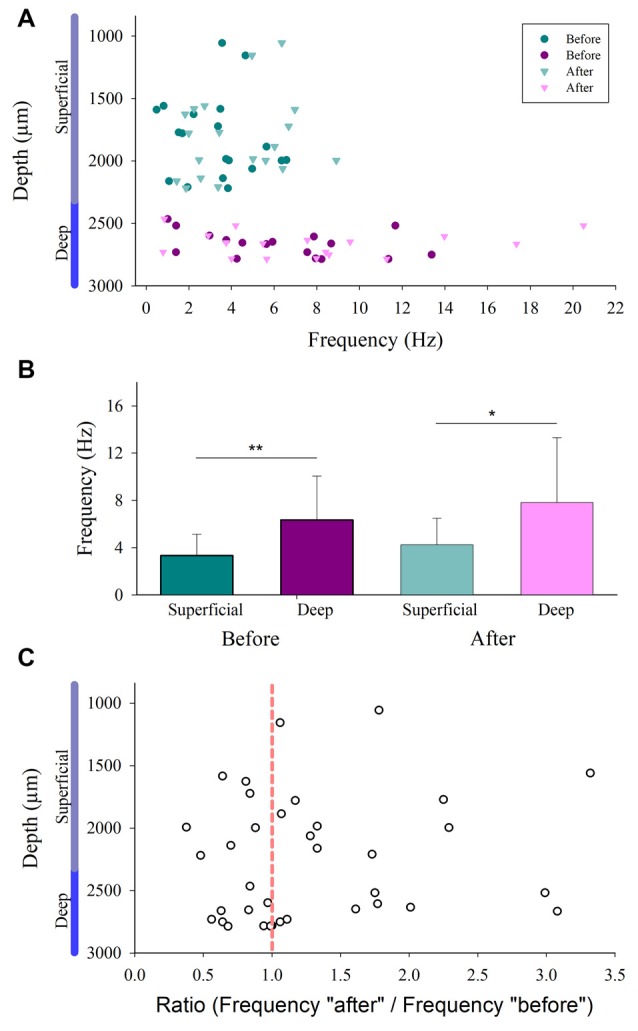
Firing rate distribution for the superficial and the deep dorsal horn neurons. **(A)** The depth vs. the firing rate (frequency in Hz) of superficial and the deep dorsal horn neurons before and after the vibratory stimuli. **(B)** Mean firing rate of the superficial and deep dorsal horn neurons for both conditions before and after the vibratory stimuli. **(C)** The after/before ratio of firing rate for the superficial and the deep dorsal horn neurons vs. the depth in which they were recorded.

We compared the firing rate for both the superficial and the deep dorsal horn neurons. Figure 5A shows a graph of the depth vs. the firing rate (frequency) of such neurons before and after the vibratory stimuli. Note that the superficial neurons exhibit a slower firing rate and less frequency dispersion than the deep neurons. Figure 5B illustrates significant differences in the mean firing rate between the superficial and deep dorsal horn neurons for both conditions before and after the vibratory stimuli. Figure 5C shows that both groups of neurons exhibit a similar dispersion in the after/before ratio of their firing rate, irrespective of their depth.

**Figure 6:**
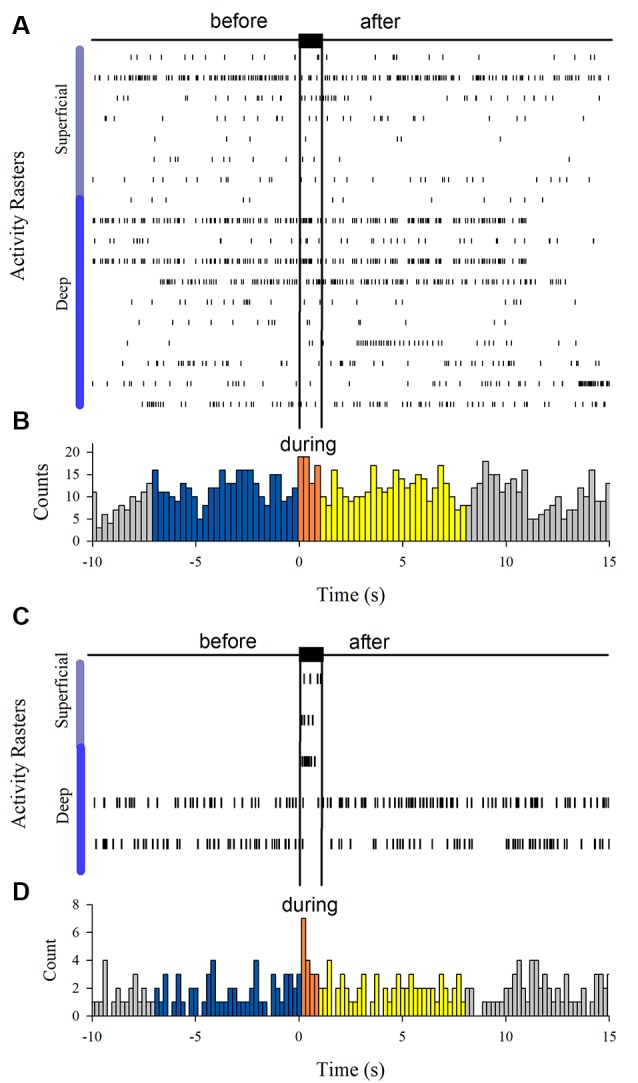
**(A)** Spiking activity of neurons not responding during the vibratory stimuli (*n* = 18). **(C)** Spiking activity of other neurons that only were excited (*n* = 3) or inhibited (*n* = 2) by the vibratory stimuli. **(B,D)** Histograms obtained from the raster displays in **(A,C)**.

Figure 6 shows the firing activity of other neurons, not exhibiting afterdischarges. We found 18 neurons that did not respond to the Ia stimulation. Moreover, we found three neurons responding to the Ia stimulation but not exhibiting sustained firing and two other neurons that were inhibited during the Ia stimulation.

## Discussion

We found interneurons in the superficial and deep dorsal horn exhibiting afterdischarges following a brief high-frequency stimulation of Ia afferents. The superficial dorsal horn neurons exhibited a significantly lower firing rate compared with the deep dorsal horn neurons. The sustained interneuronal activity of both classes of neurons was correlated to the DC potential recorded on the lumbosacral spinal cord. However, although similar stimulation schemas have been used before, in mammals (Crone et al., 1988; Hounsgaard et al., 1988; Lee and Heckman, 1998) as well as in reptiles (Hounsgaard and Kjaerulff, 1992; Russo and Hounsgaard, 1996), attention was mainly focused on the motoneuron response.

### Slow DC Potential

The amplitude of the slow DC potential distributed spatially was consistent with anatomical data about the Ia afferent branching pattern. The Ia afferents of the soleus and *gastrocnemius medialis* muscles are mainly distributed through the ipsilateral spinal segment L7 but also give numerous branches to the L6 and S1 segments (Brown and Fyffe, 1978; Ishizuka et al., 1979).

Other slow waves, as the DC potential (also termed DC shift), can be recorded in other contexts. For instance, after the pudendal nerve stimulation in the female cat, it is possible to record a “delayed depolarization” in the hind limb motoneurons that outlasted the stimulus by up to 6 s. This is consistent with our observation of a DC shift in the cord dorsum potential that also outlasted the stimulus up to 6 s (Muñoz-Martínez and Delgado-Lezama, 2007). In other studies, it was found that previous to the alternating flexor-extensor activity during scratching, a positive DC shift occurs in the hind limb enlargement of decerebrate cats (Bayev and Kostyuk, 1981). Such DC shift is correlated to the tonic activity of interneurons from the deep dorsal horn and to the postural stage that precedes scratching (Cuellar et al., 2018). As stated in our “Results” section, we recorded a similar positive DC shift correlated to interneuron activity from the deep dorsal horn. It is possible that under certain circumstances (e.g., throughout the scratch postural stage or due to high-frequency muscle spindle stimulation), interneurons could exhibit shifts in their membrane potential in a bistable-like manner. In accordance with this idea, intermediate zone neurons located in the hind limb enlargement of an *in vivo* turtle preparation go into alternating shifts in their membrane potential in a phase-dependent manner during the scratch cycle (Berkowitz et al., 2006).

Recently, it was demonstrated that in mammals, intraspinal DC stimulation leads to the facilitation of several phenomena that outlasts the period of stimulation, including the monosynaptic reflex and the rise in excitability of cutaneous afferents (Bolzoni and Jankowska, 2015; see also Jankowska, 2017). Interestingly, in our *in vivo* preparation, both the GL-ENG activity and slow DC potential also exhibited facilitation after the brief high-frequency stimulation of Ia afferents. Therefore, we cannot exclude that the observed responses are due in part to the rise in excitability of muscle afferents or to the associated K+ release, among other factors, as potentially suggested by the DC potentials. In this context, it is essential to mention some limitations of our study.

### Limitations

The phenomenon described here is a first necessary step to highlight the importance of the afterdischarges in interneurons in spinal circuits. However, several elements will need to be addressed. First, we do not know the identity of these spinal interneurons other than that they receive Ia afferent inputs and that they are distributed in the superficial and deep dorsal horn. However, they may belong to a class of neurons exhibiting plateau potentials, which could be modulated by neuromodulators, as seen in motoneurons. This could be possible because there is a wide plethora of evidence suggesting that a plateau potential is a typical process inherent to afterdischarges in spinal motoneurons and interneurons (Russo and Hounsgaard, 1996; Svirskis and Hounsgaard, 1997; Abbinanti et al., 2012; Reali and Russo, 2013; Husch et al., 2014) and in other cell types of the nervous system (Llinás and Sugimori, 1980; Flatman et al., 1983). Also, there is evidence of the postnatal emergence of serotonin-induced plateau potentials in commissural interneurons of the mouse spinal cord (Abbinanti et al., 2012) and in adult spinal V2a interneurons (Husch et al., 2014). However, more experimental evidence is needed for unveiling the electrophysiological and pharmacological nature of the dorsal horn neurons exhibiting afterdischarges. Second, we do not know the underlying mechanisms in the relationship between the DC potential in the cord dorsum vs. the possible plateau potentials in interneurons. We can only speculate in this respect based on results from other animal preparations. For instance, turtle ventral horn interneurons seem not to depend on neuromodulator actions since neither serotonin receptor agonists nor other modulators need to be added to *in vitro* cord preparations to promote plateau potentials in these cells (Hounsgaard and Kjaerulff, 1992). Similarly, dorsal horn interneurons do not rely on neuromodulators for afterdischarge generation (Russo and Hounsgaard, 1996) as opposed to motoneurons in comparable settings (Perrier and Hounsgaard, 2003). Plateaus in both interneurons and motoneurons are highly sensitive to dihydropiridines, a phenomenon that suggests common mechanisms (Russo and Hounsgaard, 1996; Morisset and Nagy, 1999; Perrier and Hounsgaard, 2003).

### Unitary Interneuronal Activity

We found that both the slow DC potential and the afterdischarge spiking activity of the spinal interneurons from the superficial and deep dorsal horn outlasted the duration of the brief high-frequency stimulation of Ia afferents around 6 s. We can compare this long-lasting activity with the activity of other cell types activated by sensory stimulation. For instance, spinal interneurons and motoneurons can exhibit similar sustained discharges after a brief exposure to electrical stimulation to the sensory pudendal nerve of female cats that outlasted the stimulus by up to 6 s (Muñoz-Martínez and Delgado-Lezama, 2007). Also, there is evidence that several types of interneurons can sustain long-lasting activity in the dorsal and ventral horns (Currie and Stein, 1990; Russo and Hounsgaard, 1994, 1996). Compared to other cell types, spinal neurons exhibit afterdischarges during especially prolonged periods: even in the tens of seconds scale. In the rodent cerebellar cortex, Purkinje cells undergo down-to-up transitions in their simple spike firing rate either spontaneously (Llinás and Sugimori, 1980) or in response to climbing fiber input. In this last case, after such an abrupt increase, the firing rate decays smoothly during a narrow 1 s time window (Loewenstein et al., 2005). In the cat, a neuronal population from the reticular nucleus of the thalamus exhibits plateau potentials during the occurrence of sleep spindles, about 1–2 s (Fuentealba et al., 2005). Cat hind limb motoneurons are said to be “fully bistable” whenever self-sustained firing lasts at least 3 s (Lee and Heckman, 1998), although there are reports of afterdischarges lasting as long as 1 min (Crone et al., 1988).

Functionally, particular interest has been paid to the so-called wide dynamic range (WDR) neurons. In the cat, WDR cells respond to low- as well as to high-threshold afferent stimulation (Salter and Henry, 1990). It is believed that WDR afterdischarges produce both secondary pain and non-nociceptive aftersensations (Price et al., 1978; Morisset and Nagy, 1998, 1999). The Ia information also reaches WDR neurons; however, its influence over these cells is mainly inhibitory (Pomeranz et al., 1968; Le Bars, 2002). Thus, we could exclude the involvement of this kind of cell in the phenomenon exposed here.

Other possible candidates that are able to sustain long-lasting activity are the diverse types of interneurons that reside in the intermediate zone, which respond to muscle stretch (Harrison and Jankowska, 1985), and laminae V–VII interneurons that establish direct excitatory and inhibitory contacts with ipsilateral motoneurons (Bannatyne et al., 2009; Jankowska, 2015). We can speculate that the afterdischarges of excitatory interneurons would reinforce the effect of motoneuron intrinsic properties to keep active the already recruited units. It must be mentioned that a reverberating loop of interneuronal activity was proposed early besides the motoneuron intrinsic properties to explain sustained motor activity (Hultborn et al., 1975). Contrarily, afterdischarges of inhibitory interneurons would tend to finish plateau in motoneurons. Additionally, inhibitory input would contribute to reverse the “normal” order of the recruitment of motor units (Heckman and Binder, 1993).

Also, in *in vitro* turtle preparations, interneurons have been found within laminae VII and VIII with plateau potentials, which emit contralateral projections (Hounsgaard and Kjaerulff, 1992). In this last case, a role during alternating motor activities was suggested. Further characterization is needed to understand whether the cat interneurons exhibiting afterdischarges also emit contralateral projections.

In conclusion, interneurons of the superficial and deep dorsal horn in the cat remain active in a parallel manner to motoneurons after ipsilateral hind limb Ia stimulation. These neurons could be characterized by the stimulation of different segmental and descending afferents, as well as by the intrinsic properties measured from intracellular recordings; however, such types of experiments are challenging in our *in vivo* preparation in cats and are out of the scope of the present manuscript. We can only show the phenomenon of afterdischarges in dorsal horn neurons that are highly correlated to a DC slow potential at the cord dorsum. Morphological and physiological characterization of these cells would be of paramount importance for a deeper understanding of the functional role of the present phenomenon.

## Data Availability Statement

The raw data supporting the conclusions of this article will be made available by the authors, without undue reservation, to any qualified researcher.

## Ethics Statement

The animal study was reviewed and approved by Ethics committee (CICUAL) from the Benemérita Universidad Autónoma de Puebla.

## Author Contributions

EM and AF conceived and designed the experiments. EM and AM-F wrote the article. AM-F, MM-C and EM performed the experiments. AM-F, MM-C, NH, AF and EM performed the analysis. All of the authors revised and approved the manuscript.

## Conflict of Interest

The authors declare that the research was conducted in the absence of any commercial or financial relationships that could be construed as a potential conflict of interest.
